# Cytochrome P450-mediated antiseizure medication interactions influence apoptosis, modulate the brain BAX/Bcl-X_L_ ratio and aggravate mitochondrial stressors in human pharmacoresistant epilepsy

**DOI:** 10.3389/fphar.2022.983233

**Published:** 2022-08-22

**Authors:** Chaitali Ghosh, Rosemary Westcott, Emilio Perucca, Mohammed Hossain, William Bingaman, Imad Najm

**Affiliations:** ^1^ Department of Biomedical Engineering, Cerebrovascular Research, Lerner Research Institute, Cleveland Clinic, Cleveland, OH, United States; ^2^ Department of Biomedical Engineering and Molecular Medicine, Cleveland Clinic Lerner College of Medicine of Case Western Reserve University, Cleveland, OH, United States; ^3^ Department of Medicine (Austin Health), The University of Melbourne, Melbourne, VIC, Australia; ^4^ Australia and Department of Neuroscience, Monash University, Melbourne, VIC, Australia; ^5^ Charles Shor Epilepsy Center, Neurological Institute, Cleveland Clinic, Cleveland, OH, United States

**Keywords:** cytochrome P450, blood-brain barrier, epilepsy, drug interaction, apoptotic signaling, pharmacodynamics, SIRT3

## Abstract

Polytherapy with antiseizure medications (ASMs) is often used to control seizures in patients suffering from epilepsy, where about 30% of patients are pharmacoresistant. While drug combinations are intended to be beneficial, the consequence of CYP-dependent drug interactions on apoptotic protein levels and mitochondrial function in the epileptic brain remains unclear. We examined the interactions of ASMs given prior to surgery in surgically resected brain tissues and of three ASMs (lacosamide, LCM; oxcarbazepine, OXC; levetiracetam LEV) in isolated brain cells from patients with drug-resistant epilepsy (*n* = 23). We divided the patients into groups–those who took combinations of NON-CYP + CYP substrate ASMs, NON-CYP + CYP inducer ASMs, CYP substrate + CYP substrate or CYP substrate + CYP inducer ASMs–to study the 1) pro- and anti-apoptotic protein levels and other apoptotic signaling proteins and levels of reactive oxygen species (reduced glutathione and lipid peroxidation) in brain tissues; 2) cytotoxicity at blood-brain barrier epileptic endothelial cells (EPI-ECs) and subsequent changes in mitochondrial membrane potential in normal neuronal cells, following treatment with LCM + OXC (CYP substrate + CYP inducer) or LCM + LEV (CYP substrate + NON-CYP-substrate) after blood-brain barrier penetration, and 3) apoptotic and mitochondrial protein targets in the cells, pre-and post-CYP3A4 inhibition by ketoconazole and drug treatments. We found an increased BAX (pro-apoptotic)/Bcl-X_L_ (anti-apoptotic) protein ratio in epileptic brain tissue after treatment with CYP substrate + CYP substrate or inducer compared to NON-CYP + CYP substrate or inducer, and subsequently decreased glutathione and elevated lipid peroxidation levels. Further, increased cytotoxicity and Mito-ID levels, indicative of compromised mitochondrial membrane potential, were observed after treatment of LCM + OXC in combination compared to LCM + LEV or these ASMs alone in EPI-ECs, which was attenuated by pre-treatment of CYP inhibitor, ketoconazole. A combination of two CYP-mediated ASMs on EPI-ECs resulted in elevated caspase-3 and cytochrome c with decreased SIRT3 levels and activity, which was rescued by CYP inhibition. Together, the study highlights for the first time that pro- and anti-apoptotic proteins levels are dependent on ASM combinations in epilepsy, modulated via a CYP-mediated mechanism that controls free radicals, cytotoxicity and mitochondrial activity. These findings lead to a better understanding of future drug selection choices offsetting pharmacodynamic CYP-mediated interactions.

## 1 Introduction

Multiple antiseizure medications (ASMs) are frequently used to control difficult-to-treat seizures in patients with epilepsy (Kwan et al., 2011). When polytherapy is used, clinically important drug-drug interactions can occur, many of which involve inhibition or induction of cytochrome P450 (CYP) isoenzymes (Patsalos et al., 2002; Perucca, 2005a; Perucca, 2005b; Perucca and Meador, 2005; Zaccara and Perucca, 2014). It is generally assumed that clinically relevant metabolic drug interactions occur in the liver and other major organs responsible for drug clearance. However, there increasing evidence that CYP enzymes in the brain also have significant biological consequences (Miksys and Tyndale, 2002; Ghosh et al., 2016). Unlike in the liver, brain CYP is concentrated near ASM targets, and can influence drug metabolism locally (Miksys and Tyndale, 2002; Gervasini et al., 2004; Ferguson and Tyndale, 2011; Anna Haduch et al., 2013; Ghosh et al., 2016; Williams et al., 2019). Many ASMs are either CYP substrates (i.e., lacosamide) or CYP inducers (i.e., oxcarbazepine), while others are not mediated by CYP at all (i.e., levetiracetam) (Contin et al., 2004; Andreasen et al., 2007; Cawello et al., 2012; McGrane et al., 2018). This poses a risk for drug-drug interactions between co-prescribed ASMs based on their involvement with CYP enzymes.

One promising third-generation ASM, for example, is lacosamide (LCM), which is metabolized by CYP enzymes. When administered alone, LCM has been shown to be neuroprotective, reduce oxidative stress and neuroinflammation, and decrease inflammation-induced cortical apoptosis in aged rats (Savran et al., 2019). Although, it is often co-prescribed with other ASMs for the treatment of epilepsy. In 2008, LCM was approved as an adjunctive therapy in adults or adolescents with epilepsy for the treatment of partial-onset seizures with or without secondary generalization in the United States and European Union (Bauer et al., 2017) and LCM monotherapy was approved as a treatment for focal epilepsy. LCM has been found to be a relatively well-tolerated medication in these patients, and the pharmacokinetic interactions of LCM with other ASMs has been described in the past years (Shandra et al., 2013; Markoula et al., 2014). However, the pharmacodynamic effects of LCM polytherapy with other ASMs, including CYP inducers and those not mediated by CYP, have not yet been described.

In general, CYP inhibition is commonly known to elevate the blood level of concomitant drugs, which can result in serious adverse reactions to the medications (Markoula et al., 2014; Sun et al., 2020). In contrast, CYP induction with combination therapy could cause decreased levels of drug in the blood, often reducing drug efficacy (Markoula et al., 2014; Sun et al., 2020); although, not much is known about the pharmacodynamic consequences elicited by interactions between CYP inducers and substrates. One case report describing the effects of oxcarbazepine (OXC), although reported to cause mild hepatic CYP induction, co-administered with other CYP substrate to treat mood disorders exhibited some worsening of psychiatric symptoms after the inducing agent was added (Baird, 2002). Another study in rats showed that combination therapy of diazepam (CYP substrate) with either phenobarbital (CYP inducer) or phenytoin (CYP inducer) caused increased neuronal apoptosis compared to monotherapy with any of these medications (Bittigau et al., 2002). ASMs can also interact with various mitochondrial pathways, structures, or functions (Berger et al., 2010; Finsterer and Scorza, 2017), affect enzymatic cascades, such as the respiratory chain, oxidative phosphorylation, or non-respiratory chain pathways, including the tricarboxic cycle or the β-oxidation pathways, and influence apoptosis associated with changes in cellular dynamics (Finsterer and Scorza, 2017). These evidences provide a glimpse into the possibility of harmful pharmacodynamic effects of ASM polytherapy, like increased apoptosis, relating to CYP involvement, which needs to be further elucidated.

Our previous *in vitro* work with endothelial cells from human epileptic brain tissue showed changes in CYP activity could result in a wide-array of molecular and cellular changes, including changes in the permeability of the blood-brain barrier (BBB) (Ghosh et al., 2015). In studies conducted in surgically resected brain specimens from patients with medically intractable epilepsy, we also found that brain CYP3A4 activity varied depending on type of ASM combinations taken by the patients, and that these changes correlated with seizure frequency (Williams et al., 2019). In the current study, we investigated resected brain tissues and isolated brain cells from medically refractory epilepsy patients treated with different ASM combinations before surgery, to identify comparative treatment effects on 1) pro-apoptotic (BAX) and anti-apoptotic (Bcl-X_L_) protein levels and other apoptotic signaling proteins and mitochondrial function implicated in cellular stress (e.g., ERK, phospho-ERK, BAD, caspase-3, cytochrome c and SIRT3) and reactive oxygen species (e.g., reduced glutathione and lipid peroxidation) in epileptic brain tissues; 2) cytotoxicity in endothelial cells from human epileptic brain tissue (EPI-ECs) and in human epileptic BBB *in vitro*, and changes in mitochondrial membrane potential in the neuronal cultures post-treatment; 3) mechanisms of mitochondrial oxidative stress in EPI-ECs focusing on the activity of SIRT3, a nicotinamide adenine dinucleotide-dependent deacetylase and a key component of mitochondria. We further assessed apoptotic and mitochondrial protein targets in EPI-ECs and normal human neuronal cells, pre- and post-CYP3A4 inhibition by ketoconazole with ASM treatments to determine the involvement of CYP enzymes in the process.

## 2 Materials and methods

### 2.1 Human subjects

Brain tissue specimens from individuals (*n* = 23) with pharmacoresistant epilepsy were obtained following focal surgical resections, using a protocol approved by the Cleveland Clinic Institutional Review Board (IRB 07-322) in compliance with the principles outlined in the Declaration of Helsinki. The epileptic nature of the resected brain tissue was identified based on prior noninvasive (scalp video-EEG monitoring, magnetic resonance imaging, positron emission tomography) and invasive (stereo-electroencephalography) investigations. Demographic details and information on seizure frequency, duration of epilepsy, age, gender, localization of the resected epileptic tissue, underlying ASM treatment, and pathology of each specimen is provided in Supplementary Table S1. Results of tests conducted on resected human epileptic brain tissues were compared based on type of ASMs taken patients at the time of surgery and at least 3 months prior to surgery. These are either ASMs as combinations of NON-CYP + CYP substrate, NON-CYP + CYP inducer, CYP substrate + CYP substrate or CYP substrate + CYP inducer. The experimental design is outlined in Supplementary Figure S1.

### 2.1 Protein isolation and Western blot analysis

Small portions of the snap-frozen resected epileptic brain tissue were homogenized in radioimmunoprecipitation assay buffer (Sigma-Aldrich, United States) with protease inhibitor (Sigma-Aldrich, United States). The tissue suspension was centrifuged at 14000G (Avanti-J25I, Beckman Coulter, United States), the supernatant was collected and the concentration of protein was measured by the Bradford method. BAX, Bcl-X_L_, ERK1/2, Phospho-ERK (Thr980), SIRT3, BAD, cytochrome c and caspase-3 were separated by 10% sodium dodecyl sulfate polyacrylamide gel electrophoresis and later transferred to polyvinylidene fluoride (PVDF) membranes (EMD Millipore Corp., Billerica, MA, United States) in semi-dry transfer (trans-Blot™ SD, Bio-Rad, United States) and blocked for 4 h at room temperature. In brief, the membranes were incubated overnight with primary antibody (see target proteins listed in Supplementary Table S2A) and subsequently probed with the appropriate secondary antibody (Supplementary Table S2B) as previously described (Williams et al., 2019; Hossain et al., 2020). For the target proteins, the PVDF membranes were incubated in stripping buffer at room temperature for 30 min followed by blocking, or a fresh gel was simultaneously repeated with the samples. Western blots were run in duplicates. In each case, the protein expression was normalized by β-actin (as a loading control) and quantified by ImageJ software.

### 2.3 Reduced glutathione assay

Reduced glutathione (GSH) was measured using a Glutathione Assay kit from Abnova (catalog KA1649) according to the manufacturer’s instructions. In this assay, 5, 5′-dithiobis (2-nitrobenzoic acid) reacts with reduced glutathione to form a yellow product. The optical density, measured at 412 nm, is directly proportional to the glutathione concentration in the sample. GSH in tissue extracts was quantified from calibration curves generated using a standard. All standards and samples were measured in duplicate. Data were normalized for protein content (Lindenmaier et al., 2005; Ghosh et al., 2015).

### 2.4 Lipid peroxidation malondialdehyde assay

Oxidative stress was assessed by quantifying lipid peroxidation with a malondialdehyde (MDA) Assay Kit, Abnova (catalog KA3736), according to the manufacturer’s instructions. Lipid peroxidation forms MDA and 4-hydroxynonenal, with the end products of the reaction providing a widely accepted measure of oxidative damage. MDA in tissue samples reacts with thiobarbituric acid to generate the MDA-thiobarbituric acid adduct which is analyzed colorimetrically (λ = 532 nm). MDA in tissue extracts was quantified from calibration curves generated using a standard (Lin et al., 2021), and data were normalized for protein content.

### 2.5 Cell culture

#### 2.5.1 Human epileptic brain endothelial cells

Primary epileptic endothelial cells were obtained from brain specimens resected from patients with drug-resistant epilepsy (*n* = 10) mostly due to focal cortical dysplasia, according to a previously described procedure (Ghosh et al., 2018; Hossain et al., 2020). Briefly, surgical specimens were incubated with collagenase type II (2 mg/ml; Worthington Biochemical Corp., Lakewood, NJ, United States) at 37°C for 20 min to dissociate the endothelial cells. The collagenase was then washed off with medium (1.5 g/100 ml, MCDB 105 supplemented with endothelial cells growth supplement 15 mg/100 ml, heparin 800 U/100 ml, 10% fetal bovine serum, and 1% penicillin/streptomycin). Cells were stained positive for von Willebrand factor and negative for glial fibrillary acidic protein (data not shown). EPI-ECs were initially expanded in 75 cm^2^ flasks pre-coated with fibronectin, 3 μg/cm^2^ (Ghosh et al., 2018; Hossain et al., 2020).

#### 2.5.2 Human astrocytes and neuronal cell culture

Normal human astrocytes (catalog 1800) were purchased from ScienCell Research Laboratories, Inc. (Carlsbad, California) and cultured in poly-D-lysine pre-coated flasks (3 μg/cm^2^) with appropriate media (Ghosh et al., 2018; Hossain et al., 2020). Human dopaminergic neuronal cells (DAN) derived from fetal brain tissues were purchased from ClonExpress (catalog number: DAN 020; Gaithersburg, MD, United States) using the culture media recommended by the manufacturer (Ghosh et al., 2015; Ghosh et al., 2022). The neuronal cultures were characterized with anti-human microtubule associated protein 2 (MAP-2) immunohistochemistry (data not shown).

#### 2.5.3 Blood-brain barrier *in vitro* system for direct evaluation of antiseizure medication access to cultured neurons

The *in vitro* BBB setup was established using flow-based *in vitro* modules (FiberCell Systems Inc., New Market, Maryland; catalog C2025) as previously described (Ghosh et al., 2018; Hossain et al., 2020). In the FiberCell polysulfide plus cartridge, each module contains 20 hollow-fiber capillaries embedded inside a clear plastic chamber, which is attached to a reservoir for media circulation and connected to a pulsatile pump. EPI-ECs (4 × 10^6^/device) were seeded in the luminal side in different devices. Astrocytes (3 × 10^6^/device) were co-cultured in the abluminal side of the *in vitro* device. BBB formation was evaluated by trans-endothelial electrical resistance measurement (Supplementary Figure S2). Different ASMs combinations were perfused through the capillaries and their effects following their passage across the BBB were directly tested on the neuronal culture present in the chamber. Specifically, passage across the BBB and subsequent effects was tested after perfusion with the non-CYP substrate levetiracetam (LEV) alone, and the CYP substrate lacosamide (LCM) alone and in combination with LEV or with a CYP inducer (oxcarbazepine, OXC). These drug treatments were chosen based on the drug regimen of this cohort of patients (see Figure 2D). The overall experimental design is outlined in Supplementary Figure S1.

The neuronal cultures exposed to ASMs through the BBB were subsequently evaluated to determine: 1) cytotoxicity by adenylate kinase measurement; 2) mitochondrial membrane potential by Mito-ID analysis; 3) levels of apoptosis and mitochondrial protein targets such as BAX, Bcl-X_L_, ERK1/2, phospho-ERK (Thr980), SIRT3, cytochrome c in the neuronal cell lysates by 10% gel electrophoresis and analyzed by Western blot. Parallel evaluations were performed to test the effects of the ASMs on EPI-ECs derived from epileptic brain tissue to determine: 1) cytotoxicity by adenylate kinase measurement; 2) mitochondrial SIRT3 activity; and 3) the apoptotic and mitochondrial protein targets such as BAX, Bcl-X_L_, ERK1/2, phospho-ERK (Thr980), SIRT3, cytochrome c, caspase-3 separated by 10% gel electrophoresis and analyzed by Western blot.

### 2.6 Adenylate kinase/toxilight cytotoxicity assay

Cytotoxicity was assessed in EPI-ECs and neuronal cells (DAN) by measuring the levels of adenylate kinase in the supernatant. This highly sensitive assay measures adenylate kinase (catalog number: LT07-217; Lonza) released from damaged mammalian cells and provides an accurate determination of the degree of cytolysis (Ghosh et al., 2015). Bioluminescent adenylate kinase is present in all cells, and loss of cell integrity through damaged membrane results in its leakage into the surrounding medium. Adenylate kinase measurements are plotted as relative luminescent units, so that approximately equal numbers of cells (6 × 10^5^ cells/per chamber) exposed to different drug exposures can be compared with the control and the 0 h time point. Cytotoxicity measurements were made with and without pretreatment with the CYP3A4 inhibitor ketoconazole (KCZ, 10 µM) for 2 h, followed by exposure to ASMs alone or in combination (LCM, LEV, LCM + LEV, LCM + OXC).

### 2.7 Mitochondrial membrane potential

Mitochondria play a central role in cellular metabolism, bioenergetics and apoptosis (Zorova et al., 2018). The Mito ID^®^ Membrane Potential Cytotoxicity Kit measures fluctuations in mitochondrial membrane potential utilizing a cationic dual-emission dye that exists as green fluorescent monomers in the cytosol, and accumulates as orange fluorescent J-aggregates in the mitochondria. The formation of JC-1 monomers and their fluorescence are linearly correlated with the decrease in membrane potential. Briefly, neuronal cells were incubated with JC-1 at 37°C for 15 min. The Mito ID kit (catalog ENZ-51019-KP002, Enzo Life Sciences Inc., NY, United States) was used for the assay. Mitochondria having a low membrane potential accumulate low concentrations of dye and exhibit a green fluorescence, while more highly polarized mitochondria produce an orange signal. The effect of ASMs alone (LCM; LEV) and combined (LCM + OXC; LCM + LEV) were tested to evaluate mitochondrial membrane potential changes (ΔΨm) in the neuronal cells. Carbonyl cyanide m-chlorophenylhydrazone (CCCP) was used as positive control to determine uncoupled mitochondria. The Bio-Tek Synergy fluorescence microplate reader was used to measure the signal. This photophysical property is due to the reversible formation of J-aggregates upon membrane polarization that causes shifts in emitted light from ∼530 nm (the emission of the monomeric dye) to 590 nm (the emission of the J-aggregate form) when excited at 490 nm. The percentage mitochondrial membrane potential signal was compared with a 4 µM CCCP concentration as recommended by the manufacturer.

### 2.8 SIRT3 activity assay

The deacetylase activity of SIRT3 in EPI-ECs (*n* = 3) exposed to ASMs alone (LCM or LEV) or combined (LCM + OXC; LCM + LEV) was measured by a fluorometric method using the SIRT3 Activity Assay Kit (catalog ab156067, Abcam, Waltham, MA United States). The manufacturer’s protocol and recommendations were followed. We tested in the same EPI-ECs (*n* = 3) the effect of CYP inhibition SIRT3 activity after exposure to the ASM alone and combined. Briefly, the enzymatic reaction was initiated by incubating 15 µg of protein from test samples (prepared without using protease inhibitor) with 20 µM of a SIRT3 specific fluoro-substrate peptide, 200 µM of NAD and 5 µL of developer solution. Three control experiments were performed, one without cell lysate, one without the recombinant enzyme and one without NAD. A standard curve for SIRT3 expression was fitted using 0, 400, 800 and 1,000 ng of recombinant SIRT3. Deacetylase activity was evaluated by measuring the fluorescence at 2 min time intervals for 60 min using a CYTATION/5 Microplate reader (BioTek Instruments, United States) (Michalak et al., 2017; Yi et al., 2019). Fluorescent intensity was plotted against each time-point to follow the enzyme activity, and relative SIRT3 activity was determined by the mean end-point measurements and compared between the groups.

### 2.9 Protein isolation from cell lysate and Western blot after antiseizure medication treatment post-CYP inhibition

Both cell types (EPI-ECs and DAN) in each treatment group were cultured separately in 100 mm Petri dishes in standard/recommended medium. At 80% confluency, cells were exposed to ASMs alone (LCM; LEV) and combined (LCM + OXC; LCM + LEV), with and without 2 h-KCZ pre-treatment. After 24 h of ASM exposure, cells were lysed using lysis buffer with protease inhibitor cocktail for Western blot analysis. Cell lysates were separated through a 10% SDS gel and transferred onto PVDF membranes. The membranes were blocked and incubated with primary antibodies against BAX, Bcl-X_L_, BAD, ERK1/2, Phospho-ERK (Thr980), SIRT3, cytochrome c, caspase-3 or β-actin overnight at 4°C. The next day, the membranes were incubated with specific secondary antibodies for 1 h at room temperature as described previously (Ghosh et al., 2015; Williams et al., 2019). The antibody details are provided in Supplementary Table S2.

### 2.10 Statistical analysis

All data are expressed as mean ± standard error of the mean (SEM). The statistical analysis were conducted based on the applied test for comparison of data sets as appropriate by two-sample *t*-test. One-way or two-way analysis of variance (ANOVA) followed by a Tukey post hoc test was used for multiple comparison. A *p* value of <0.05 was considered statistically significant. Origin 9.0 software (OriginLab Corp., Northampton, MA, United States) was used for the statistical analyses.

## 3 Results

### 3.1 BAX and Bcl-X_L_ imbalance in epileptic brain tissue based on type of antiseizure medication exposure

**FIGURE 1:**
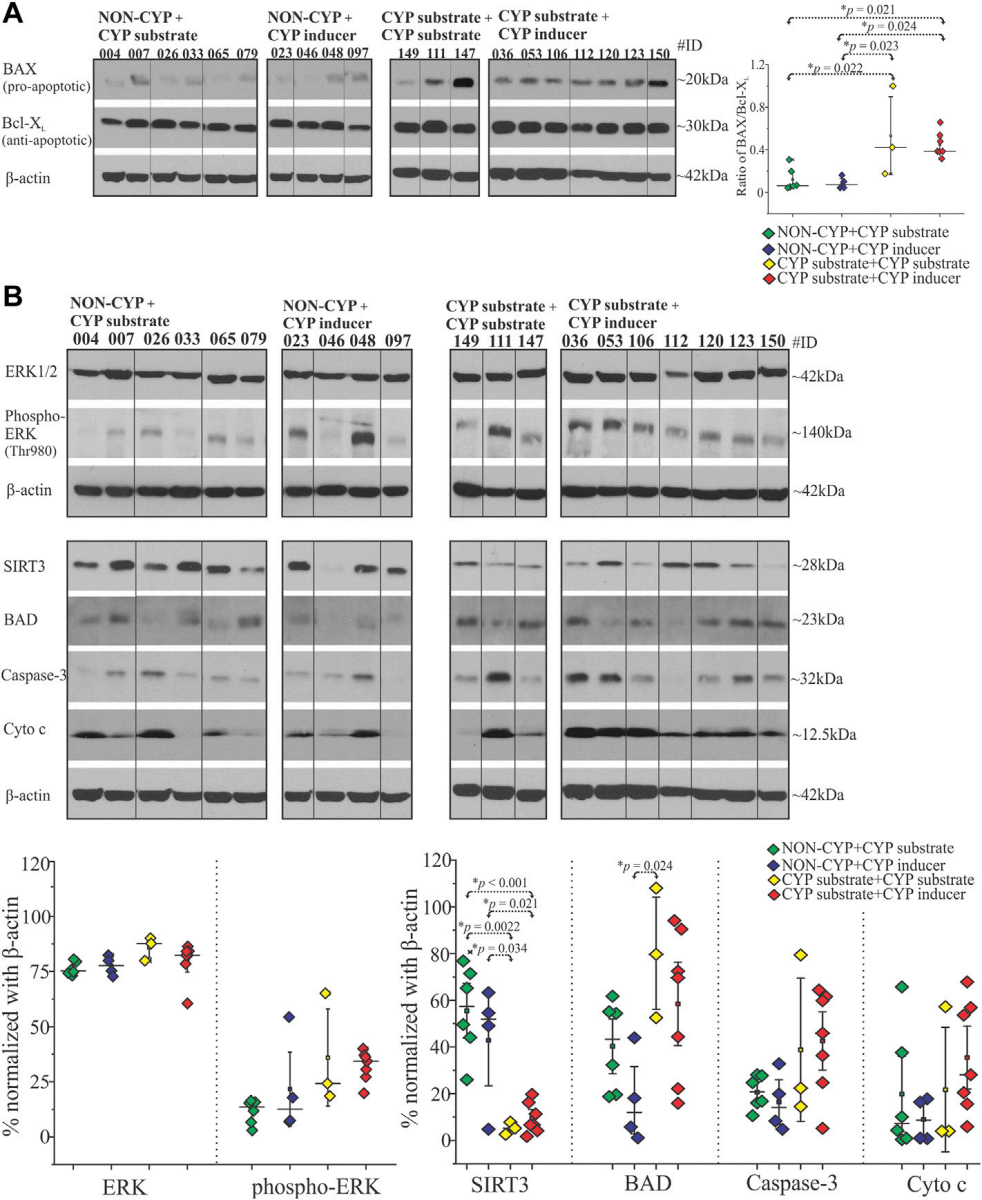
Antiseizure medications as CYP-substrates or inducers or in combination with NON-CYP-mediated ASMs alters the BAX and Bcl-X_L_ levels, affects other apoptotic and mitochondrial target proteins in epileptic brain. **(A)** BAX (pro-apoptotic) and Bcl-X_L_ (anti-apoptotic) analysis of the brain tissue shown by a representative western blots (*n* = 10 subjects/group) and the densitometric quantification normalized with β-actin were used to depict the ratio of BAX/Bcl-X_L_ levels plotted to compare within the groups. **(B)** A significant decrease in mitochondrial deacetylase SIRT3 levels was observed in the CYP substrate + CYP substrate or CYP inducer groups compared to the NON-CYP + CYP substrate or CYP inducer groups in epileptic brain tissues (total *n* = 20 subjects). BAD protein levels are significantly increased in the CYP substrate + CYP substrate group compared to the NON-CYP + CYP inducer group. Data are normalized with β-actin. Results are expressed as mean ± SEM by ANOVA.

We analyzed the samples from a patient cohort categorized by type of ASM treatment taken and evaluated the pre-characterized epileptic tissue by histopathological (post-resection) staining as described previously (Williams et al., 2019). The snap-frozen epileptic brain tissues from twenty patients divided into four groups based on their ASM treatment regimen before surgery: NON-CYP + CYP substrate, NON-CYP + CYP inducer, CYP substrate + CYP substrate and CYP substrate + CYP inducer. The western blot analysis showed that in the CYP substrate + CYP substrate (*p = 0.022; *p = 0.023) and CYP substrate + CYP inducer (*p = 0.021; *p = 0.024) groups the BAX (pro-apoptotic) to Bcl-X_L_ (anti-apoptotic) ratio were significantly elevated compared to the NON-CYP + CYP substrate and NON-CYP + CYP inducer groups, respectively (Figure 1A). This finding is indicative of higher pro-apoptotic condition in epileptic brain tissue of patients receiving a combination of CYP substrates or CYP substrates with CYP inducers. The full blots are shown in Supplementary Figure S3.

### 3.2 Alterations in apoptotic and mitochondrial signaling proteins based on type of antiseizure medication exposure

Beside BAX and Bcl-X_L_, other key apoptotic and mitochondrial signaling proteins were assayed in epileptic brain tissue obtained from patients in the NON-CYP + CYP substrate, NON-CYP + CYP inducer, CYP substrate + CYP substrate and CYP substrate + CYP inducer groups and compared. ERK1/2 expression did not differ significantly between the groups (Figure 1B). However, phosphorylated ERK (Thr980) levels showed an elevated trend in the CYP substrate + CYP inducer group compared to the NON-CYP + CYP substrate group (*p* = 0.08). Compared to the NON-CYP + CYP substrate and NON-CYP + CYP inducer groups, epileptic brain tissues from the CYP substrate + CYP inducer group showed an increasing trend in levels of BAD (*p* = 0.59 and 0.085, respectively), although not significant (Figure 1B). The cytochrome c and caspase-3 levels within the patients groups did not show a significant difference. However, BAD levels were significantly increased (*p = 0.024) in the CYP substrate + CYP substrate group compared to the NON-CYP + CYP inducer group. In contrast, compared to the NON-CYP + CYP substrate and NON-CYP + CYP inducer groups, a significant decrease in SIRT3 protein levels, depicting mitochondrial dysfunction in the epileptic brain tissues, is noticed across individuals that received a combination of ASMs as CYP substrate + CYP substrate (*p = 0.0022 and 0.034, respectively) or CYP substrate + CYP inducer (*p < 0.001 or *p = 0.021, respectively).

### 3.3 Oxidative stress related to CYP combination of antiseizure medications

**FIGURE 2:**
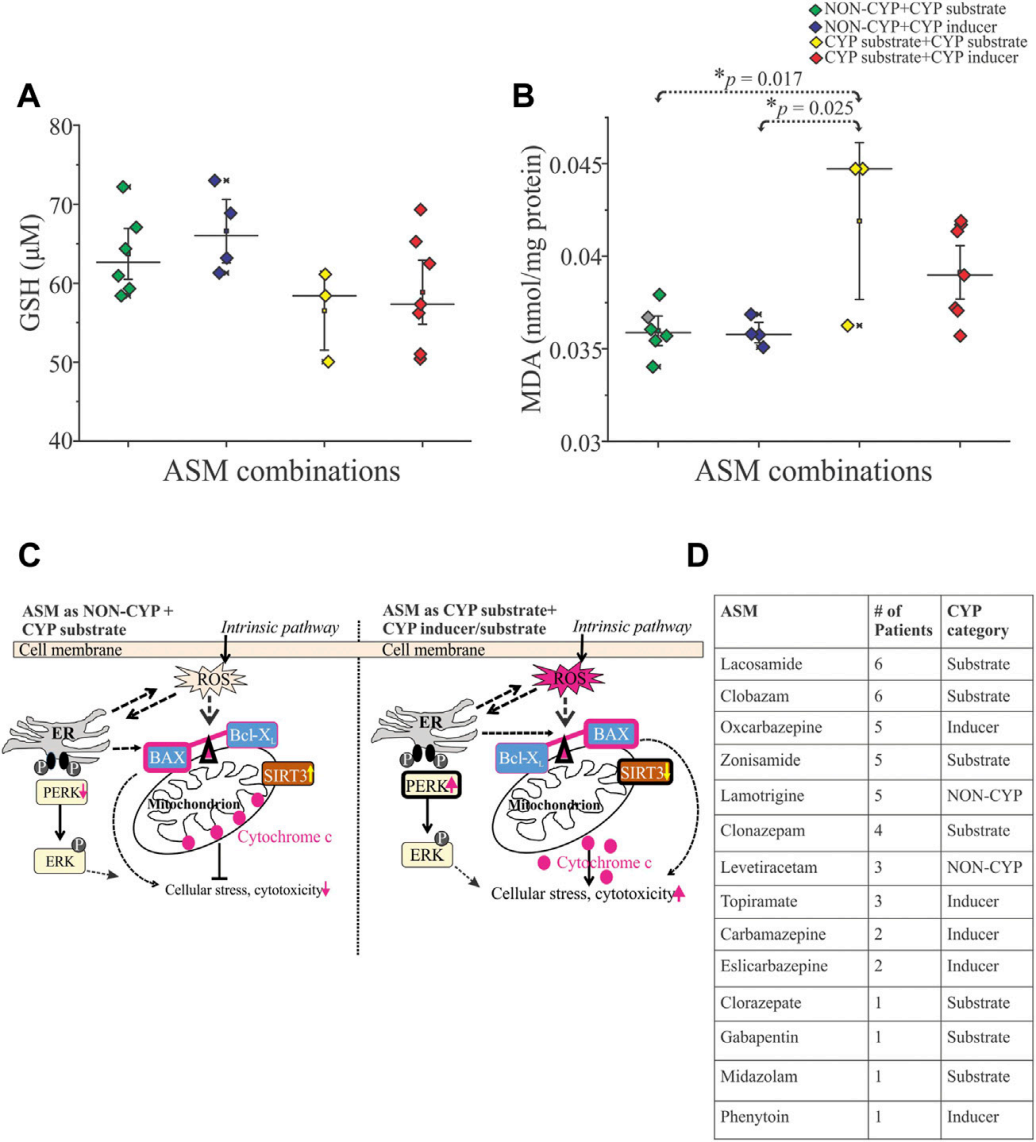
Reactive oxygen species influenced by ASM combinations based on CYP association in human epileptic brain tissue. **(A,B)** Cytotoxicity associated with oxidative stress (*n* = 20 subjects total), as shown by decreased trend of reduced glutathione GSH, **(A)** and significantly increased levels of free radical showed by malondialdehyde MDA, **(B)** indicative of increased lipid peroxidation in patients taking multiple CYP substrates compared to the NON-CYP + CYP substrate (*p = 0.017) or inducer groups (*p = 0.025) (*n* = 20 subjects total). Results are expressed as mean ± SEM by ANOVA. **(C)** Schematic scheme showed two potential scenarios based on the data generated in the human epileptic brain tissues based on ASM combination given before surgery as NON-CYP + CYP substrate vs. CYP substrate + CYP inducer/substrate (in combination) causing cellular distress. Release of reactive oxygen species (ROS), potentially imbalance the BAX (pro-apoptotic)-Bcl-X_L_ (anti-apoptotic) ratio linking to endoplasmic reticulum (ER) phospho-ERK expression contributing to cellular stress via cytochrome c upregulation and mitochondrial SIRT3 downregulation with majorly CYP-mediated ASMs treatments. **(D)** Table describing the distribution of ASMs taken by this cohort of patients (*n* = 20) and whether they are a CYP substrate, CYP inducer or NON-CYP-mediated.

Evidence that the cytotoxic effects were mediated by production of free radicals is provided by measuring reduced glutathione and malondialdehyde (MDA) levels in these epileptic brain tissues. There was no significant difference in reduced glutathione levels between the NON-CYP + CYP substrate, NON-CYP + CYP inducer, CYP substrate + CYP substrate and CYP substrate + CYP inducer groups (Figure 2A). Although, there was a significant increase in MDA levels (Figure 2B), a measure of lipid peroxidation (marker of oxidative stress), in the CYP substrate + CYP substrate group compared to patients taking ASMs as NON-CYP + CYP substrate (*p = 0.017) or NON-CYP + CYP inducer (*p = 0.025). In contrary, the patients that received ASMs as NON-CYP + CYP substrate or inducer, in combination showed relatively increased levels of reduced glutathione and lower MDA levels, indicating negligible oxidative stress.

### 3.4 Cytotoxicity and mitochondrial membrane potential influenced by antiseizure medications at blood-brain barrier epileptic endothelial cells and neuronal cells

**FIGURE 3:**
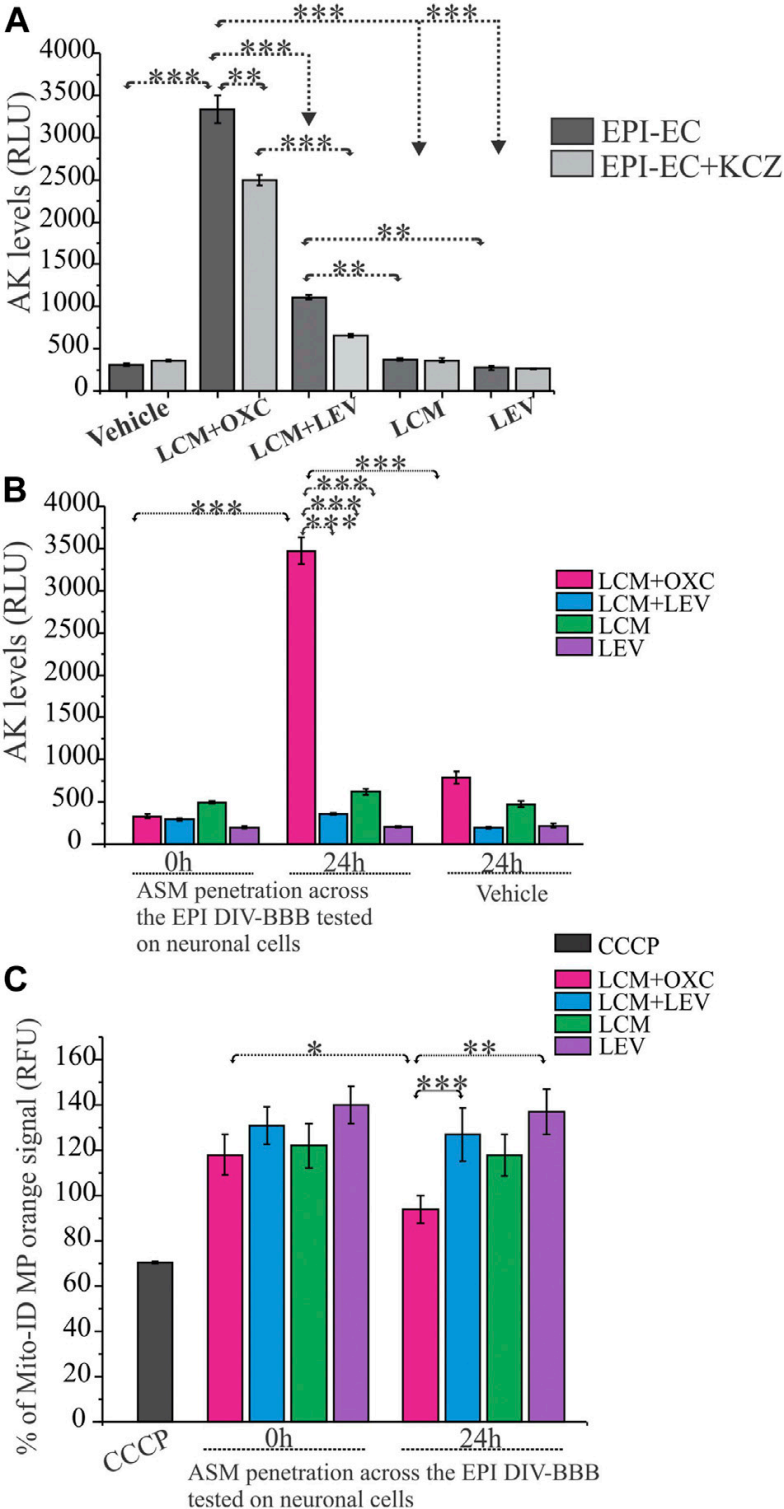
CYP-mediated neurotoxicity across the *in vitro* epileptic BBB affects mitochondrial function. **(A,B)** Cytotoxicity after 24-h exposure to LCM + OXC vs. LCM + LEV, LCM or LEV alone on EPI-ECs and neuronal culture after BBB penetration was associated with increased levels of adenylate kinase (AK) released from damaged cells, measured in relative luminescence units (RLU). The elevated LCM + OXC (*p < 0.001) induced cytotoxicity **(A)** and neurotoxicity **(B)** compared to LCM + LEV, LCM, LEV or vehicle was measured by adenylate kinase levels and could be prevented in EPI-ECs **(A)** by pre-treatment with CYP inhibitor, ketoconazole (KCZ) for 2 h before ASM co-treatment (*p < 0.01). **(C)** LCM + OXC co-exposure at the BBB significantly decreased Mito-ID levels in the neuronal cells (measured by orange RFU levels) at 24 h compared LCM + LEV (*p < 0.001) together or LEV (**p < 0.01) alone. Also, the Mito-ID alterations was significant (*p < 0.05) in 0–24 h, post-LCM + OXC co-treatment. CCCP positive control was used as an indicator for mitochondrial damage tested directly on human neuronal culture. Quantification of adenylate kinase levels **(A–B)** and % Mito-ID membrane potential (MP) are depicted as mean ± SEM by two-way ANOVA, ***p < 0.001, **p < 0.01, *p < 0.05.

A functional blood-brain barrier protects the brain from circulating neurotoxins. In these experiments, the EPI-ECs derived from the epileptic brain tissues (*n* = 3 each) were exposed to ASM combinations as LCM + OXC (CYP substrate + CYP inducer), LCM + LEV (CYP substrate + NON-CYP) or ASM alone, LCM (CYP substrate) or LEV (NON-CYP) to determine if ASM combination based on CYP-mediated pathway together or alone can exert toxicity at epileptic brain endothelial cells. Further, the blood-brain barrier penetrants from the brain-side of the *in vitro* blood-brain barrier were used for neurotoxicity evaluation on normal primary human dopaminergic neuronal cells. As shown in Figure 3A, the LCM + OXC-induced cytotoxic response on EPI-ECs was significantly elevated compared to LCM + LEV (***p < 0.001) in combination, LCM (***p < 0.001) or LEV (***p < 0.001) alone determined by adenylate kinase levels. Whether the cytotoxicity in EPI-EC observed was due to drug interactions mediated by CYP enzymes was further validated by pre-treatment with CYP inhibitor, ketoconazole, and followed by drug treatment. A significant reduction in cytotoxicity and decreased adenylate kinase levels (Figure 3A) of LCM + OXC (**p < 0.01) in EPI-ECs were observed with ketoconazole, compared to EPI-ECs without CYP inhibition. Similarly, exposure of drug penetrant across the epileptic *in vitro* BBB showed increased adenylate kinase levels on neuronal cells (Figure 3B) post-LCM + OXC treatment, compared to non-CYP ASMs (***p < 0.001) in combination or ASM alone, i.e., LCM (***p < 0.001) or LEV (***p < 0.001). Further, the LCM + OXC in combination also showed decreased mitochondrial membrane potential in the neuronal cells (Figure 3C) compared to LCM + LEV (***p < 0.001) or LEV alone (**p < 0.01) at 24 h.

### 3.5 Decreased mitochondrial SIRT3 activity in EPI-ECs in LCM + OXC or LCM improved with CYP inhibition

**FIGURE 4:**
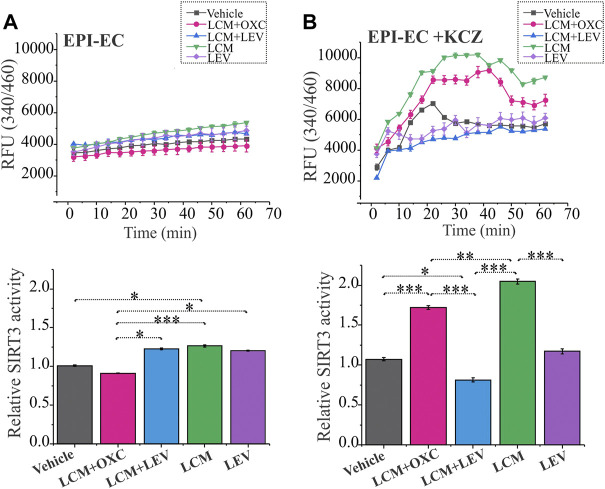
Improved SIRT3 activity with CYP-inhibition in human EPI-ECs post-LCM + OXC treatment. **(A)** SIRT3 activity showed a decreased level (depicted by relative fluorescent units, RFU) over time post-LCM + OXC co-treatment compared LCM + LEV, LCM or LEV, with the relative levels at 60 min quantified below. **(B)** CYP inhibition with ketoconazole showed significant elevation in the SIRT3 activity in LCM + OXC and LCM alone compared to LCM + LEV or LEV alone suggesting an association of CYP-mediated drugs (in combination or alone) and mitochondrial activity change in EPI-ECs. Results are expressed as mean ± SEM by one-way ANOVA was performed with a Tukey *post hoc* test, *p < 0.05, **p < 0.01, ***p < 0.001.

Mitochondrial SIRT3 activity in EPI-ECs was assessed after treatment with ASMs in combinations (LCM + OXC; LCM + LEV) and ASM alone (LCM; LEV) were evaluated to determine CYP-dependent drug interactions. EPI-ECs exposed to LCM + OXC in combination showed relatively low SIRT3 activity (Figure 4A) compared to LCM + LEV in combination (*p < 0.05) or LCM alone (***p < 0.001) or LEV alone (*p < 0.05). A similar trend was observed in vehicle-treated EPI-ECs that showed decreased levels of relative SIRT3 activity when compared to LCM alone. Although, the time-course of SIRT3 activity for 60 min with drug treatment were also plotted to identify the relative pattern with time which shows negligible difference overtime.

To further confirm the alteration in the mitochondrial dynamics is due to CYP involvement in the EPI-ECs, we pretreated the EPI-ECs for 24 h with CYP inhibitor, ketoconazole, then followed with the ASM regimen. EPI-ECs pretreated with ketoconazole showed upregulation of SIRT3 activity after LCM + OXC in combination or LCM alone when compared to LCM + LEV in combination (***p < 0.001) or LEV alone (***p < 0.001) showing a distinct differential time-course pattern followed for 60 min under each condition (Figure 4B). Ketoconazole seems to be beneficial for EPI-ECs as the vehicle alone (*n* = 3) post ketoconazole treatment (without ASM) also showed a significant increase (*p < 0.05) in SIRT3 activity when compared to LCM + LEV in combination.

### 3.6 CYP inhibition replenishes the levels of apoptotic and mitochondrial signaling proteins influenced by CYP-dependent antiseizure medication interactions in EPI-ECs and neuronal cells

**FIGURE 5:**
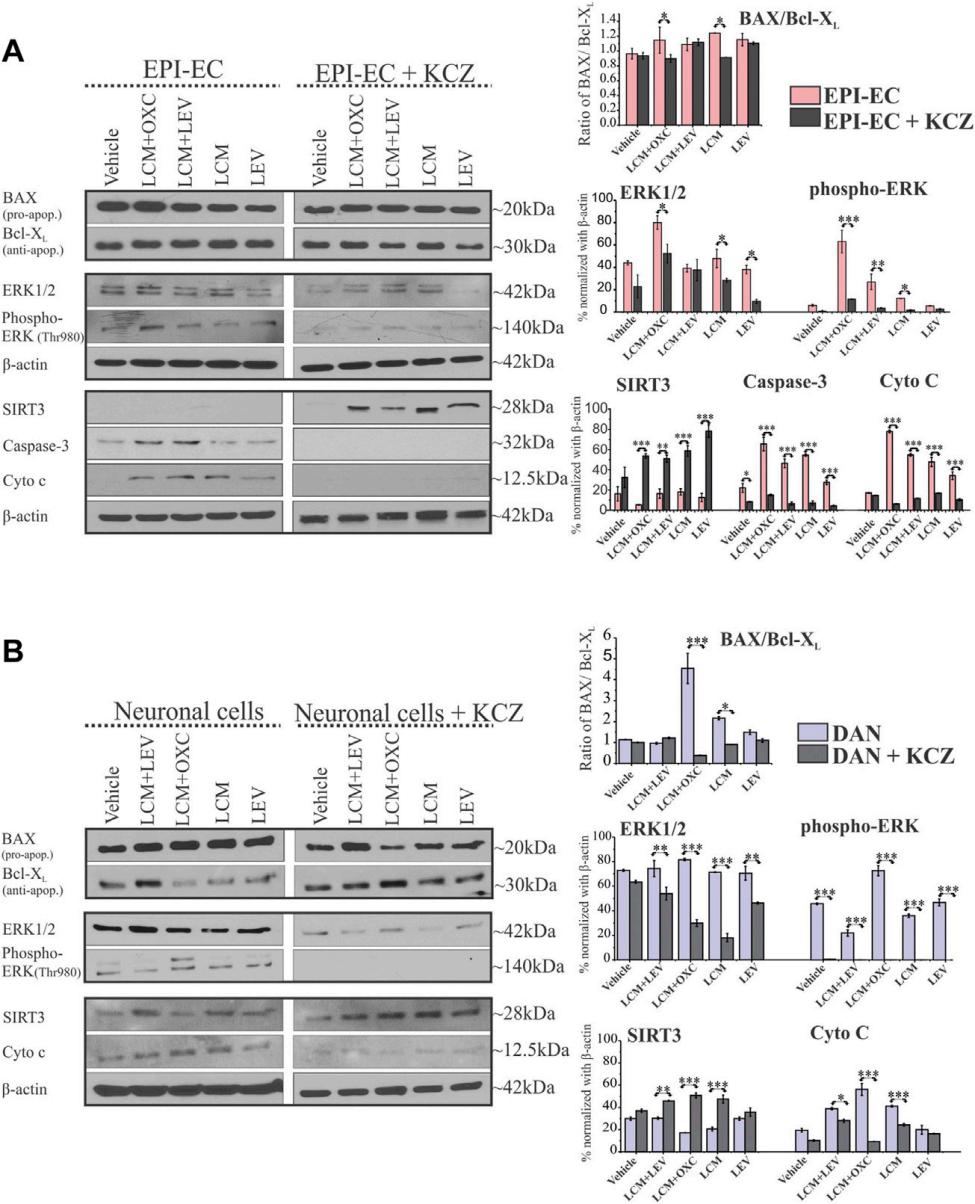
CYP inhibition causes a selective decrease in apoptotic proteins and elevated SIRT3 levels in human EPI-ECs and neuronal cells. **(A,B)** Western blot showed a significant decrease in the BAX (pro-apoptotic)/Bcl-X_L_ (anti-apoptotic) ratio in LCM + OXC (*p < 0.05) and LCM (*p < 0.05) expression in EPI-ECs **(A)** and in LCM + OXC (***p < 0.001) and LCM (*p < 0.05) expression in neuronal cells **(B)**, post-CYP inhibition with ketoconazole (KCZ) within the same cell types. Similarly, the levels of ERK1/2 (*p < 0.05), phospho-ERK (***p < 0.001, LCM + OXC and *p < 0.05, LCM), caspase-3 (*p < 0.001) and cytochrome c (***p < 0.001) showed a significant decrease post-CYP inhibition, markedly in LCM + OXC co-treated and LCM alone group in EPI-ECs along with improved SIRT3 (***p < 0.001) levels. Likewise in neuronal cells **(B)**, a similar pattern was seen with significantly decreased levels of ERK1/2 (**p < 0.01), phospho-ERK (*p < 0.001) and cytochrome c (*p < 0.001) post-CYP inhibition in the LCM + OXC co-treated group and increased SIRT3 (*p < 0.001) levels. Western blot quantitative data (in right) are normalized with β-actin, and values are plotted as mean ± SEM by two-way ANOVA with a Tukey *post hoc* test for each target, *p < 0.05, **p < 0.01, ***p < 0.001.

Consistent with the epileptic brain tissue, an elevated BAX/Bcl-X_L_ ratio in neuronal cells (*n* = 3) was noticed post-LCM + OXC (*p < 0.001) and LCM (*p < 0.001) treatment, compared to LCM + LEV and LEV alone (Figure 5B). EPI-ECs (*n* = 3) also showed upregulated BAX/Bcl-X_L_ ratio with LCM + OXC (*p < 0.001) and LCM (*p < 0.001) compared to LCM + LEV and LEV alone (Figure 5A and Supplementary Figure S4). Moreover, CYP inhibition with KCZ decreased the BAX (pro-apoptotic)/Bcl-X_L_ (anti-apoptotic) ratio in spite of follow up-drug treatment, both with LCM + OXC in combination (*p < 0.05) and LCM alone (*p < 0.05) in the same EPI-ECs (*n* = 3) (Figure 5A). Likewise, a significant decrease in the BAX/Bcl-X_L_ ratio was also seen with KCZ pre-treatment after LCM + OXC in combination (***p < 0.001) and LCM alone (***p < 0.05) in the same neuronal cells (*n* = 3) compared to drug treatment, without CYP inhibition (Figure 5B). The full blots are shown in Supplementary Figure S4.

The expression of other apoptotic signaling proteins, ERK1/2 in EPI-ECs treated with LCM + OXC compared to LCM + LEV (*p < 0.001), or LCM (*p < 0.001), or LEV (*p < 0.001), or vehicle (*p < 0.001) was found to be upregulated (Figure 5A). Similarly, phospho-ERK levels were consistently elevated within LCM + OXC co-treatment compared to LCM + LEV (*p < 0.001) or LCM (*p < 0.001) or LEV (*p < 0.001). The levels of other cellular stressors, such as cytochrome c, were also high after LCM + OXC co-treatment compared to LCM + LEV co-treatment (*p < 0.001), or LCM (*p < 0.001), or LEV (*p < 0.001), or vehicle (*p < 0.001) in EPI-ECs, and so was the increased caspase-3 levels in EPI-ECs when exposed to LCM + OXC and compared to LCM + LEV (*p < 0.001) in combinations, or LCM (*p < 0.001). Although, the condition reversed with CYP inhibitor, ketoconazole pretreatment (Figure 5A) as levels of ERK1/2 (*p < 0.001), phospho-ERK (*p < 0.001), cytochrome c (*p < 0.001), caspase-3 (*p < 0.001) decreased significantly after co-treatment of LCM + OXC thereafter in EPI-ECs. Also, within these EPI-ECs LCM + OXC or LCM + LEV co-treatment or LCM or LEV also, showed significant reduction in caspase-3 (*p < 0.001) and cytochrome c (*p < 0.001) levels with CYP inhibition (Figure 5A).

Similarly in neuronal cells, the phospho-ERK levels (Figure 5B) were subsequently elevated with LCM + OXC co-treatment compared to LCM + LEV in combination (*p < 0.001) or LCM (*p < 0.001) or LEV (*p < 0.001) alone. Other cellular stressor, such as cytochrome c levels were also increased in the same cells, after LCM + OXC co-treatment compared to LCM + LEV (*p < 0.001), or LCM (*p < 0.001), or LEV (*p < 0.001), or vehicle (*p < 0.001) treatment. Consistent to EPI-ECs, the condition were reversed with CYP inhibitor, ketoconazole in neuronal cells (Figure 5B), as levels of ERK1/2 (*p < 0.001), phospho-ERK (*p < 0.001), cytochrome c (*p < 0.001) were subsequently decreased after LCM + OXC co-treatment. In addition, CYP inhibitor, ketoconazole (Figure 5B) also decreased the levels of ERK1/2 (*p < 0.05), phospho-ERK (*p < 0.05) and cytochrome c (*p < 0.001) after LCM treatment in the neuronal cells.

It is also noteworthy that CYP inhibition with ketoconazole improved mitochondrial SIRT3 levels after LCM + OXC (***p < 0.001), LCM + LEV (***p < 0.001), LCM (***p < 0.001) and LEV (***p < 0.001) treatment compared to ASM(s) without (Figure 5A). Similarly, elevated SIRT3 levels in neuronal cells with ketoconazole pre-treatment was also identified after co-treatment with LCM + OXC (***p < 0.001), LCM + LEV (***p < 0.001), LCM (***p < 0.001) and LEV (***p < 0.001) treatment, when compared to ASM(s) without (Figure 5B) showing CYP-mediatory effect on ASM response.

## 4 Discussion

Polytherapy is common in patients with epilepsy, particularly among those with drug-resistant seizures (Patsalos et al., 2002; Perucca and Meador, 2005; Gidal et al., 2009), potentially leading to clinically-relevant drug interactions (Patsalos and Perucca, 2003). This study pinpoints for the *first time* that LCM in combination with certain other ASMs, like OXC as a CYP inducer, affect the pro- and anti-apoptotic protein levels in the epileptic brain, modulated via a CYP-mediated mechanism that controls free radicals, cytotoxicity and mitochondrial activity. The main goal of this study was therefore to identify the functional consequence of CYP-mediated ASM interactions on apoptotic signaling proteins and assessing mitochondrial function in human epileptic brain tissues, focusing at the neurovascular interface. Additionally, the two-ASM approach could aid in delineating the regulatory mechanisms of LCM with and without other CYP-dependent ASMs using alternative metabolic and regulatory pathways, facilitating improved drug efficacy via mitigating drug-induced cellular stress.

### 4.1 Increased BAX/Bcl-X_L_ ratio, BAD, phosphorylated ERK, mitochondrial dysfunction and cellular stress in human epileptic brain tissues linked to CYP-mediated drug interactions

We identified that patients taking a combination of multiple ASMs with varying CYP involvement–NON-CYP + CYP substrate, NON-CYP + CYP inducer, CYP substrate + CYP substrate and CYP substrate + CYP inducer–showed differential effects on the ratio of pro-apoptotic (e.g., BAX) and anti-apoptotic (e.g., Bcl-X_L_) proteins in the epileptic brain tissues. Evidence supports that both BAX and Bcl-X_L_ genes/proteins are expressed in the neocortical region of temporal lobe epilepsy (Henshall et al., 2000). Beside, an aberrant expression pattern of BAX and Bcl-X_L_ has been reported to participate in several other neurological disorders including epilepsy with focal cortical dysplasia and hippocampal sclerosis (McMasters et al., 2000; Chamberlain and Prayson, 2008; Toscano et al., 2019). In focal cortical dysplasia type IIb, balloon cells a histopathological characteristic, have shown apoptotic protein expression in the epileptic tissue regions, with 61% of cases in this study showing >50% of balloon cells expressing BAX (Chamberlain and Prayson, 2008). BAD (Bcl-2-associated death protein) is also another pro-apoptotic factor that associates with BAX and contributes to the regulation of cytochrome c release (Niquet and Wasterlain, 2004). A study found that in a rat model of epilepsy, seizures induced the dimerization of BAD with BcL-X_L_ in the affected areas of the hippocampus (Henshall et al., 2002). In the current study, we found that CYP association in ASM combination is critical and linked to an elevation in the BAX/Bcl-X_L_ ratio and increased BAD protein levels, suggestive of more pro-apoptotic and less anti-apoptotic proteins in epileptic brain tissues exposed mostly to ASMs that are CYP-mediated (CYP substrate + CYP substrate or CYP substrate + CYP inducer) compared to NON-CYP + CYP mediated ASMs (Figure 1) within the same patient cohort. A previous report in rats showed that dexamethasone (CYP inducer) treatment increased the cytotoxic effects of an herbal supplement due to elevated reactive metabolite levels (Haouzi et al., 2000). In this current study, the combinations of CYP substrate ASMs with other CYP substrates or inducers could lead to the production of reactive metabolites, as previously touched on by our group (Ghosh et al., 2015). To uncover the consequence, the findings also indicate relatively low levels of reduced glutathione and elevated lipid peroxidation in the brain tissues, implying oxidative stress influenced by specific ASM interactions (Figure 2). Such drug interaction was reported with haloperidol and valerian, where an increase in lipid peroxidation levels and dichlorofluorescein reactive species production was observed in rat hepatic tissue (Dalla Corte et al., 2008). Further, ASM with non-ASM could also factor into these interactions in epilepsy patients with other comorbidities (Gidal et al., 2009; Bosak et al., 2019). A similar differential modulation of the BAX and Bcl-X_L_ ratio by two-drug combinations was demonstrated in studies using human colon tumor cells (McMasters et al., 2000) in the past, which was implicated in the development of cancer and related to the mechanism of cell death induced by pro-apoptotic agents. However, other forms of cell death mechanisms could also be at play, which needs to be investigated further.

Interestingly in the epileptic tissues, we evaluated the ERK1/2 as MAP kinases which are necessary for G1-to S-phase progression and are associated with the induction of positive regulators of the cell cycle and inactivation of anti-proliferative genes (Bozzi et al., 2011). ERK1/2 expression was comparable regardless of the ASM combination in both patient groups; however, the endoplasmic reticulum-related stress signified by up-trending levels of the phosphorylated form of ERK was predominant across individuals that received ASMs that are mostly CYP-mediated (either CYP substrates or with an inducer) (Figure 1). Studies show that downstream events lead to activation of caspase-3, an important contributing factor for cellular stress augmented by increased release of cytochrome c from the mitochondria (Kirkland and Franklin, 2001, 2015; Mao et al., 2019). Caspase-3 mRNA levels have been previously found to increase in a rat model of epilepsy (Sun et al., 2012), which could be even further increased in the presence of neurotoxic chemicals (Folch et al., 2010). Further, patient-to-patient variability could play a role in the caspase-3 expression spectrum observed among the patient groups. Moreover SIRT3, a vital indicator of mitochondrial respiration, was decreased in these epileptic brain tissues in individuals taking ASMs as CYP substrate + CYP substrate or CYP substrate + CYP inducer in combinations vs. NON-CYP + CYP substrate or inducer. This suggests that a medication regimen with ASMs eliminated via different drug metabolizing pathways is safer in retaining cellular and mitochondrial integrity (results summarized in Figure 2C). SIRT3 alteration is a critical indicator of reactive oxygen species generation (Figures 2A,B) and underlying cellular stress under the exogenous influence of the interactions of ASMs that are primarily dependent on CYP as a metabolizer, like LCM, in combination with other CYP substrate or CYP inducing medications. Multiple alternative apoptotic signaling pathways also may participate (Henshall and Simon, 2005), beyond pro-apoptotic and anti-apoptotic ratio in epilepsy due to ASM interactions. Another report showed that despite an increase in the Bcl-2/BAX ratio, the granular neurons and glia exhibited active caspase-3 expression in temporal lobe epilepsy hippocampi compared to controls (Toscano et al., 2019), where glial and neuronal death were found to be increased in sclerotic hippocampi, independently of hippocampal type pathology and co-localization found with gliosis. Furthermore, in the current study among the patient cohort, the patients that received ASMs as NON-CYP + CYP substrates or inducers in combination showed relatively balanced redox homeostasis with increased level of reduced glutathione and lower lipid peroxidation levels, together indicating an altered dynamic in apoptotic and mitochondrial proteins levels (schematic representation in Figure 2C). These results may stem from CYP-mediated ASM interactions linked to metabolic pathways and oxidative stress implicating a unique feature based on ASM combination, not solely a disease pathogenesis *per se.* LCM was the most commonly taken CYP-substrate ASM in this cohort of 20 patients retrospectively studied, while OXC was the most commonly taken CYP inducer (Figure 2D).

### 4.2 Drug-induced cytotoxicity and compromised mitochondrial membrane potential affecting the neurovascular interface

To gain better insight into the mechanism of how this cellular stress could specifically influence the neurovascular interface, we evaluated the impact of LCM, a CYP-associated ASM, in combination with a CYP inducer or a NON-CYP-mediated ASM at the epileptic human *in vitro* blood-brain barrier and applied the penetrants obtained directly from the abluminal or brain-side of the blood-brain barrier onto human neuronal cultures (Supplementary Figure S1). Consistent with our previous studies (Ghosh et al., 2015), we noted CYP-mediated drug interaction in this case with LCM + OXC (CYP substrate + inducer) causing significant cytotoxicity shown by increased adenylate kinase levels in EPI-ECs compared to LCM + LEV or ASM alone (Figure 3A). Earlier studies also indicated changes in epileptic and non-epileptic CYP3A4 expression in the brain, which increased in patients taking ASMs that were CYP-mediated, and was downregulated when CYP- and NON-CYP-mediated ASMs were given together. Brain CYP3A4 levels also correlated with seizure frequency within the same individuals (Williams et al., 2019). However, in the current study, CYP inhibition with ketoconazole decreased the cellular stress observed after LCM + OXC co-treatment in EPI-ECs. Similarly, the ASM interaction at the epileptic blood-brain barrier and penetrants further showed similar cellular stress on the neuronal cells, which was demonstrated by elevated adenylate kinase levels with LCM + OXC treatment (Figure 3B), consistent with previous studies with drug-induced toxicity by two CYP-mediated ASMs (Ghosh et al., 2015). Furthermore, in neuronal cells, a decrease of mitochondrial membrane potential was also noted after LCM + OXC penetration across the epileptic human *in vitro* BBB compared to LCM + LEV or LEV alone. Overall, the study suggested that the CYP-mediated cellular stress at the epileptic-blood-brain barrier is translated across the barrier into the neuronal cells, altering mitochondrial integrity post-co-treatment with ASMs that broadly undergo CYP-mediated drug metabolism.

SIRT3 dysfunction is related to several neurological conditions such as cancer, aging, metabolic disorder and neurodegenerative diseases (Weir et al., 2013; Yi et al., 2019). In our study, a decrease in SIRT3 activity was noticed in EPI-ECs post LCM + OXC co-treatment and EPI-ECs by itself (Figure 4A); however, co-treatment of LCM + LEV or LCM or LEV alone showed upregulated SIRT3 activity suggesting that CYP-mediated ASM co-treatment does not improve mitochondrial activity as supported by SIRT3 function. Nonetheless, CYP inhibition abolished the breakdown and instead improved the mitochondrial function via upregulated SIRT3 activity post-LCM + OXC co-treatment, compared to EPI-ECs untreated or treated with LCM + LEV or LEV alone (Figure 4B). This is further supported by another study which demonstrated elevation in SIRT3 prevents apoptosis by lowering reactive oxygen species and inhibiting components of the mitochondrial permeability characteristics such as the transition pore and mitochondrial deficits associated with aging and neurodegeneration could be slowed or even prevented by SIRT3 activation (Kincaid and Bossy-Wetzel, 2013). SIRT3 dysfunction is identified in a chronic epilepsy model which is linked to mitochondrial acetylation process (Gano et al., 2018). Drug combination might contribute to this process according to the current findings. ASMs could modify mitochondrial oxidative phosphorylation based on the ASM used as noted in a pilot study (Berger et al., 2010) by evaluating mitochondrial ATP production and enzymatic activities of respiratory chain complexes in children who suffer from epilepsy, treated by a single ASM (phenobarbital, carbamazepine or lamotrigine) in peripheral white blood cells and when compared with healthy non-treated individuals as control.

### 4.3 Role of CYP inhibition on BAX/Bcl-X_L_ ratio, apoptotic signaling protein and mitochondrial SIRT3 at the human epileptic brain endothelial cells and neuronal cells

Several chemotherapeutic-induced adverse side-effects are mediated by mitochondrial dysfunction. For example, trastuzumab and sunitinib toxicity is mainly associated with mitochondrial impairment and was mostly reversible (Gorini et al., 2018). However, doxorubicin-induced toxicity not only includes mitochondrial damage but can also lead to more robust and extensive cell injury which is often irreversible and lethal (Gorini et al., 2018). While evaluating the BAX/Bcl-X_L_ in the EPI-ECs and neuronal cells (Figure 5), we identified a similar alteration pattern as in the human epileptic brain tissues, with elevated BAX/Bcl-X_L_ post-treatments with LCM + OXC compared to vehicle or untreated cells. However, CYP inhibition prevented this imbalance in both cell types, analyzed as fundamental components of the neurovascular unit, with a significant decrease in the BAX/Bcl-X_L_ ratio after LCM + OXC with ketoconazole pre-treatment. Similarly, the ERK1/2 levels were found to be elevated in EPI-ECs and neuronal cells as observed in epileptic tissues regardless of drug exposure or not but showed a significant reduction in levels post-CYP inhibition with ketoconazole. Furthermore, the phospho-ERK levels were upregulated with LCM + OXC compared to other ASMs in both EPI-ECs and neurons, as previously seen in epileptic brain tissues exposed to CYP substrates in combination with other CYP substrates or inducers before surgery. The phospho-ERK expression in these cells decreased significantly to almost negligible levels post-CYP inhibition, suggesting the beneficial role of CYP inhibitors when multiple CYP-mediated ASMs are administered, perhaps by balancing out the CYP induction with inhibition, subsequently preventing the CYP substrate from being quickly metabolized.

Downstream, caspase-3 in EPI-ECs and cytochrome c in EPI-ECs and neuronal cells post-drug treatment were similarly rescued by CYP inhibition, and a significant decrease in the protein levels was noted thereafter in these cells pre-treated with ketoconazole followed by ASM alone or ASMs together. SIRT3 consistent with epileptic brain tissues was dismantled in EPI-ECs post-ASM interaction and was reversed back post-CYP inhibition with ketoconazole. The same pattern was identified in neuronal cells. Together, the findings emphasize the relevance of cytochrome P450 enzymes via CYP overactivity that could be detrimental to cell efficacy causing cellular stress due to potential drug interactions.

## 5 Conclusion

**FIGURE 6:**
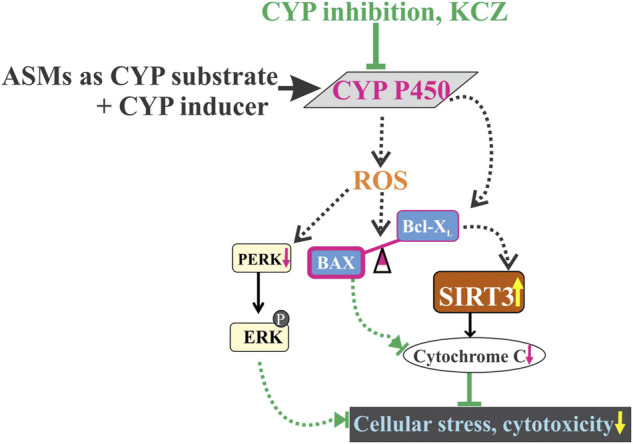
Drug-induced cellular stress by CYP-mediated drug combinations of ASMs, CYP substrate + CYP inducer co-treatment which may have contributed towards oxidative stress by reactive oxygen species (ROS) generation and aberrantly expresses an imbalance of increased BAX (pro-apoptotic) and decreased Bcl-X_L_ (anti-apoptotic) levels, possibly leading to a decrease in SIRT3 activity and expression. ROS release and increased phospho-ERK levels influence cytotoxicity at cellular levels. However, CYP inhibition completely abolished the altered cascade of apoptotic signaling pathway, thereby restoring cell integrity and mitochondrial function.

ASMs that are either CYP substrates or inducers used in combination could adversely affect tissue and cell function due to overactivity of CYP enzymes triggered by drug interactions. These interactions result in the release of reactive oxygen species and ER stress, with elevated phospho-ERK compromising cellular integrity. Blood-brain barrier EPI-ECs play an important role in CYP-mediated drug interactions controlling subsequent neuronal stress. The imbalanced BAX/Bcl-X_L_ ratio could influence the cascade of apoptotic events and consequent mitochondrial dysfunction via decreased SIRT3 activity can be rescued and reversed by CYP inhibition (Figure 6), indicating that this phenomenon involves CYP activity. Better understanding of these effects and development of new drugs that could act against novel targets could allow more rational polytherapy selection in the future.

## Data Availability

The original contributions presented in the study are included in the article/Supplementary Material, further inquiries can be directed to the corresponding author.
