# Supplementary material for: Cytochrome P450-mediated antiseizure medication interactions influence apoptosis, modulate the brain BAX/Bcl-XL ratio and aggravate mitochondrial stressors in human pharmacoresistant epilepsy
Source: Front Pharmacol. 2022 Aug 22:983233. doi: 10.3389/fphar.2022.983233 (PMC9441576; doi:10.3389/fphar.2022.983233)
Supplement: Supplementary file 1 [file DataSheet1.PDF]

Supplementary Material

Supplementary Material

1 Supplementary Figures and Tables

1.1 Supplementary Figures

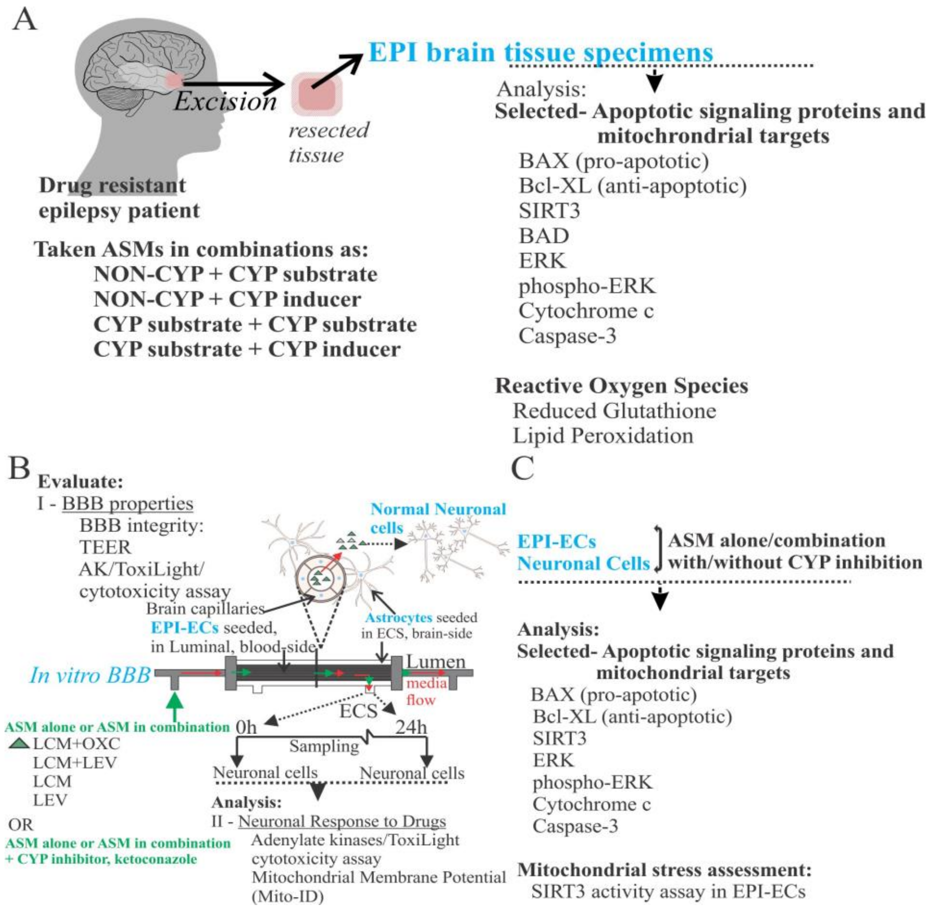

Supplementary FIGURE 1. General Experimental outline and target analysis plan. (**A**) To study the effect of ASMs in combinations as NON-CYP+CYP substrate, NON-CYP+CYP inducer, CYP substrate+CYP substrate or CYP substrate+CYP inducer given before surgery (continued at least 2-3 months until then) on epileptic human brain tissues. We studied: a) expression of apoptotic signaling proteins and mitochondrial targets, and b) levels of reactive oxygen species. (**B**) To evaluate the effect of ASM alone vs ASMs in combination, based on CYP-associated drug metabolism. Further assess the role of CYP inhibition by ketoconazole on the ASM or ASMs in combination (LCM+OXC, LCM+LEV, OXC, LEV) on human blood-brain barrier epileptic endothelial cells (EPI-ECs, derived from resected epileptic brain tissues) and on the neuronal cells (human dopaminergic neuronal cells) after penetrants were tested across the human epileptic BBB in vitro. To assess a) cytotoxicity, and b) mitochondrial membrane potential on the neuronal cells. (**C**) Evaluate the effect of ASMs alone and in combinations (LCM+OXC, LCM+LEV, OXC, LEV) on EPI-ECs and neuronal cells by evaluating, a)

the key apoptotic signaling proteins and mitochondrial targets, pre-and post CYP inhibition with ketoconazole, and b) Mitochondrial stress by measuring SIRT3 activity, pre- and post CYP inhibition on EPI-ECs.

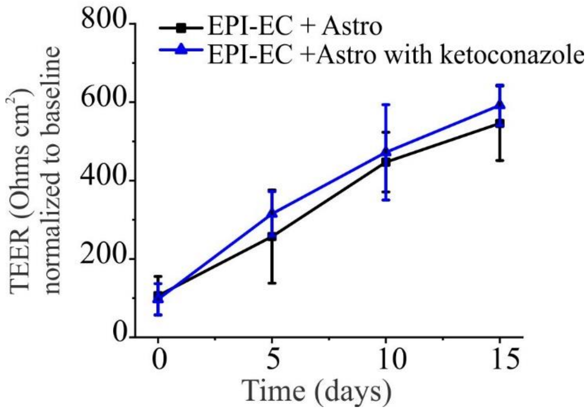

Supplementary FIGURE 2. Blood brain barrier (BBB) integrity at *in vitro* epileptic BBB with and without CYP inhibition. Transendothelial electrical resistance (TEER) showing a development of barrier with time characterized by increase TEER normalized with the baseline compared within EPI DIV-BBB, with and without CYP inhibition using ketoconazole.

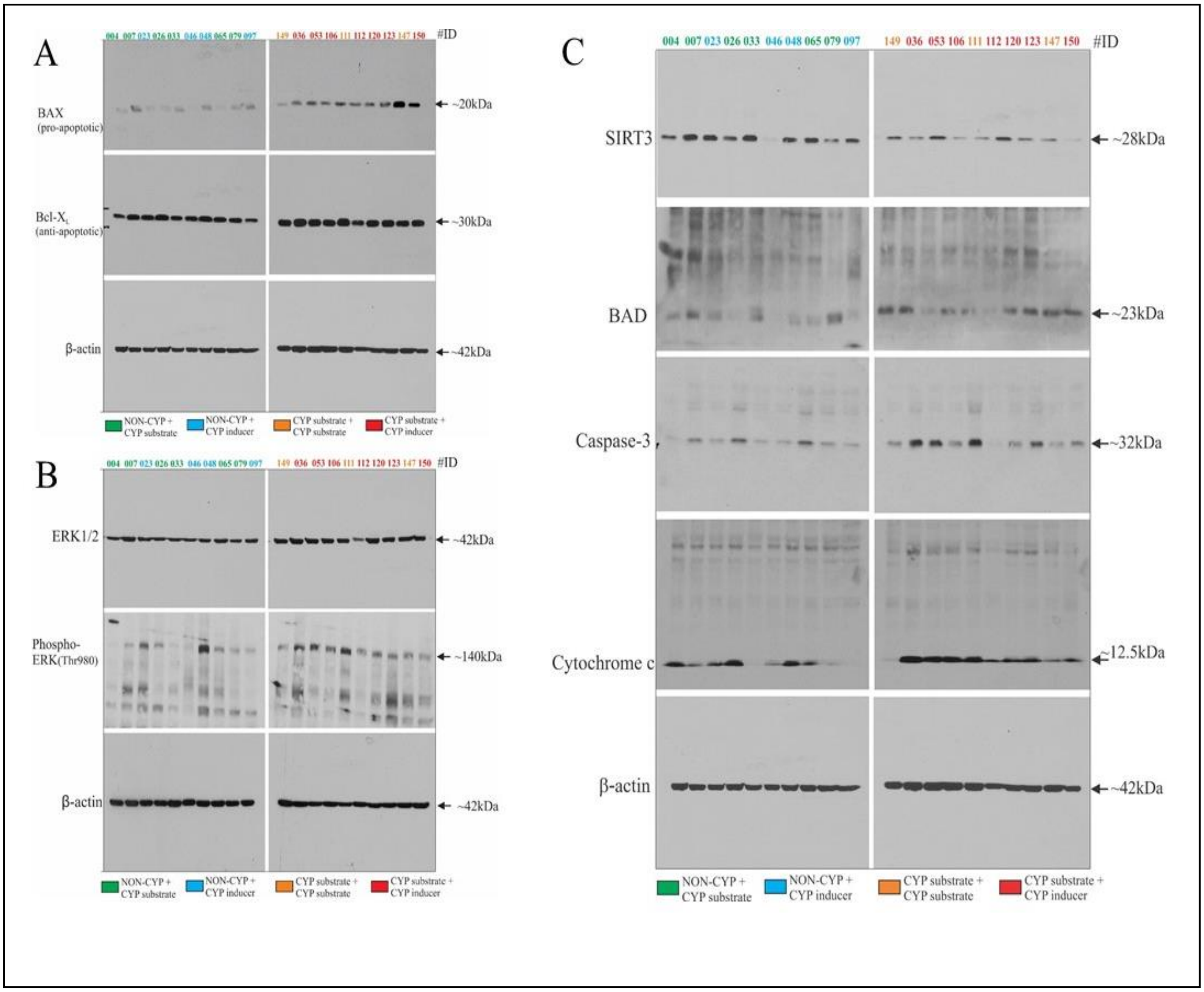

Supplementary FIGURE 3. Full western blots (A-C) for all targets analyzed in the epileptic brain tissues are presented where patients received ASM either as CYP substrates or CYP+NON-CYP substrates in combinations, before surgery. The representative blots from western blot analysis of BAX, Bcl-X_L, ERK1/2, Phospho-ERK (Thr980), SIRT3, BAD, Cytochrome c and Caspase-3 are provided with their corresponding β-actin as loading controls.

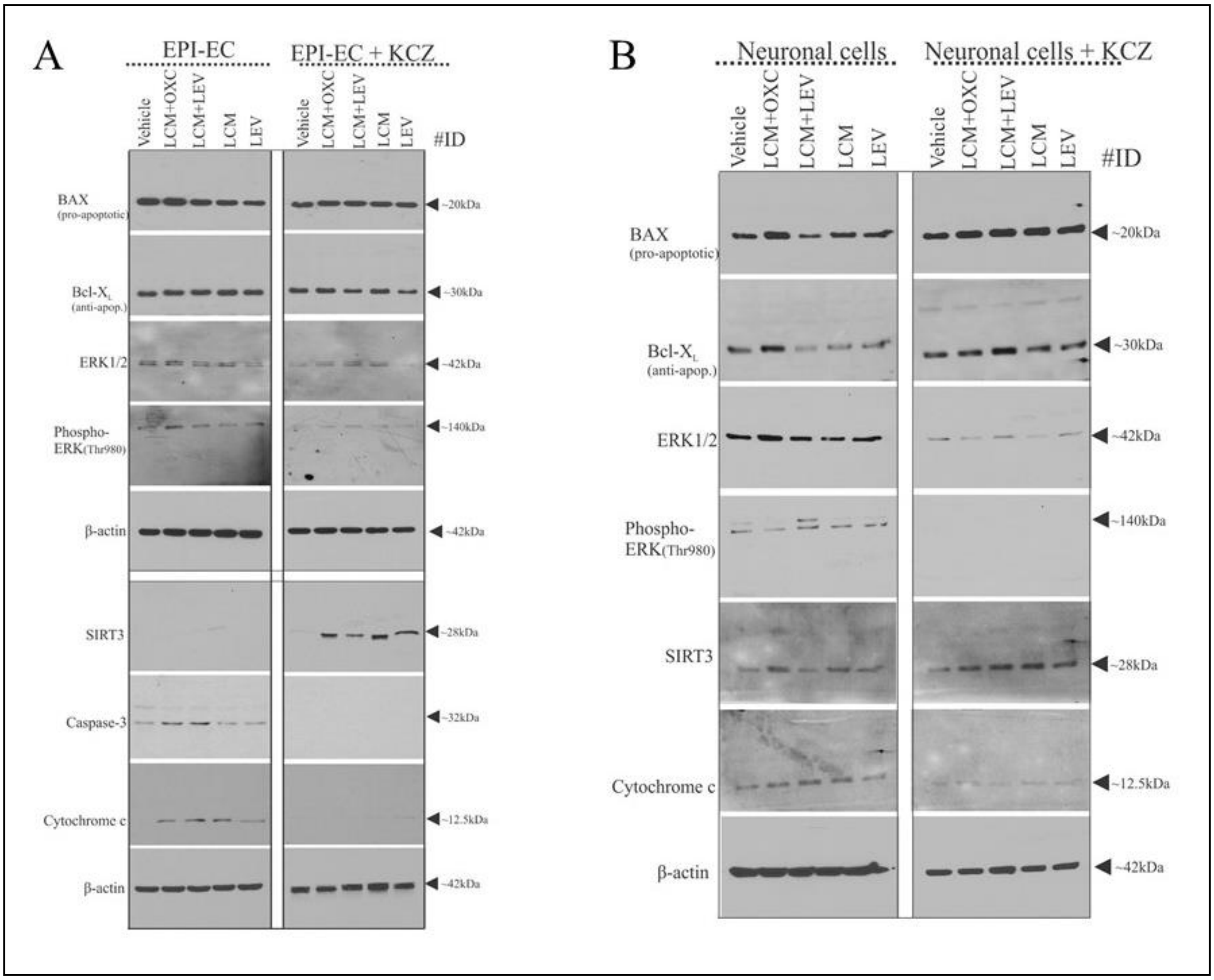

Supplementary FIGURE 4. Full western blots (A-B) for all targets analyzed in the human epileptic brain endothelial cells (EPI-ECs, A) and in the human dopaminergic neuronal cells (B) with and without drug treatments, along with CYP inhibitor ketoconazole, KCZ. The representative blots from western blot analysis of BAX, Bcl-X_L, ERK1/2, Phospho-ERK (Thr980), SIRT3, cytochrome c and caspase-3 (in EPI-ECs) are provided with their corresponding β-actin as loading controls

1.2 Supplementary Tables

1.2.1 Supplemental TABLE 1. Demographic details:

ID	Age (yrs)	Gender	ASMs	ASMs as CYP-substrate	Seizure Freq. (per week)	Duration of Epilepsy (yrs)	Resected tissue region	Pathology
4	19	M	LCM, LTG	LCM	3-4	19	Temporal lobe	FCD
7	17	F	LEV ZNS	ZNS	21	8	Left lateral Temporal lobe	FCD; subpial gliosis
23	23	M	LTG OXC	OXC	<1	9	Right frontal lobe	FCD
26	27	M	LTG ZNS	ZNS	<1	11	Left frontal lobe	Mild FCD
33	37	M	LCM LTG	LCM	1	15	Left Temporal lobe	FCD
46	35	F	LEV TPM	TPM	1	27	Right Frontal Lobe	FCD
48	36	F	OXC GBP	OXC	1	25	Mesial Left frontal lobe	FCD
65	16	M	LCM Midazolam	LCM	1	2	Left Temporal lobe	FCD

79	33	M	LEV<br>CLB	CLB	10±2	Not known	Right temporal lobe	FCD,<br>subpial gliosis
97	49	M	LTG<br>CBZ<br>PHT	CBZ<br>PHT	35±6	37	Left Temporal parietal lobe	Mild FCD
149	20	F	LCM<br>CLB	LCM<br>CLB	<1	37	Right Occipital<br>Lobe	FCD
36	45	M	ESL<br>CLB	ESL<br>CLB	<1	27	Right Temporal Lobe	FCD
53	24	M	CBZ<br>ZNS	CBZ<br>ZNS	3	11	Right Occipital<br>lobe	FCD
106	17	M	OXC<br>CLZ	OXC<br>CLZ	1±2	11	Left Frontal lobe	FCD
111	35	M	ZNS<br>CLB<br>CZP	ZNS<br>CLB<br>CZP	21±1	9	Right Temporal lobe	FCD
112	9	M	OXC<br>TPM	OXC<br>TPM	7±1	8	Right Temporal<br>Lobe	FCD
120	32	M	CLB<br>ESL<br>LCM	CLB<br>ESL<br>LCM	7±3	6	Left Lateral Temporal Lobe	FCD

123	37	F	CZP<br>TPM	CZP<br>TPM	21	12	Right Lateral Temporal	FCD
147	5	F	CLB<br>CZP<br>LCM	CLB<br>CZP<br>LCM	7	1	Left Frontal Cortex	FCD
150	10	M	ZNS<br>OXC<br>CZP	ZNS<br>OXC<br>CZP		6	Right Parietal	FCD
5	26	F	LCM<br>RFM<br>LEV<br>PHT<br>TPM	LCM<br>PHT<br>TPM	2±1	13	Right Parietal Lobe	Focal perivascular chronic inflammation,<br>focal perivascular atrophy<br>subpial gliosis
16	16	M	OXC<br>LTG	OXC	<1	14	Right Temporal Lobe	FCD
41	13	F	OXC<br>LTG<br>CLB	OXC<br>CLB	14	11	Right Frontal Lobe	FCD

Abbreviations: M: male; F: female; ASMs: antiseizure medications; LCM: Lacosamide; LEV: Levetiracetam; LTG: Lamotrigine; OXC: Oxcarbazepine; PHT: Phenytoin; CLB: Clobazam; ESL: eslicarbazepine acetate; VGB: Vigabatrin; ZNS: Zonisamide; TPM: Topiramate; CZP: Clonazepam; CBZ: Carbamazepine; RFM: Rufinamide; GBP: Gabapentin; FCD: Focal cortical dysplasia; yrs: years.

1.2.2. Supplemental TABLE 2. List of antibodies used for western blot

A

Primary Antibody	Host	Concentration	Manufacturer	Catalog No.
BAX	Rabbit	WB: 1:1000	Cell Signaling Technology, Danvers, MA	5023
Bcl-X_L	Rabbit	WB: 1:1000	Cell Signaling Technology, Danvers, MA	2764
ERK1/2	Rabbit	WB: 1:800	Abcam, Cambridge, MA	ab17942
Phospho-ERK (Thr980)	Rabbit	WB: 1:2000	Cell Signaling Technology, Danvers, MA	3192s
SIRT-3	Rabbit	WB: 1:1000	Abcam, Cambridge, MA	ab217319
BAD	Rabbit	WB: 1:800	Cell Signaling Technology, Danvers, MA	9239T
Cyto c	Mouse	WB: 1:100	Santa Cruz Biotechnology, Inc., Dallas, TX	sc13560
Caspase-3	Rabbit	WB: 1:1000	Novus Biologicals, Littleton, CO	NB100-56113
β-actin	Mouse	WB: 1:10000	Sigma-Aldrich, St. Louis, MO	A1978

B

Secondary Antibody	Concentration	Manufacturer	Catalog No.
Polyclonal Goat Anti-Rabbit IgG HRP	WB: 1:3000	Dako, part of ThermoFisher Scientific Waltham, MA	P0448
Polyclonal Goat Anti-Mouse IgG HRP	WB: 1:3000	Dako, part of ThermoFisher Scientific Waltham, MA	P0447
